# The Growing Skyline of Advanced Hepatocellular Carcinoma Treatment: A Review

**DOI:** 10.3390/ph14010043

**Published:** 2021-01-08

**Authors:** Francesca Matilde Schipilliti, Ingrid Garajová, Giulia Rovesti, Rita Balsano, Federico Piacentini, Massimo Dominici, Fabio Gelsomino

**Affiliations:** 1 Oncology Unit, University Hospital of Modena and Reggio Emilia, Largo del Pozzo 71, 41125 Modena, Italy; giulia.rovesti@gmail.com (G.R.); federico.piacentini@unimore.it (F.P.); massimo.dominici@unimore.it (M.D.); 2 Medical Oncology Unit, University Hospital of Parma, Via Gramsci 14, 43126 Parma, Italy; rita.balsano@studenti.unipr.it

**Keywords:** hepatocellular carcinoma, target therapies, immune checkpoint inhibitors, combination therapies, COVID-19

## Abstract

Hepatocellular carcinoma (HCC) is the main type of liver cancer. In the majority of cases, HCC is diagnosed at the advanced stage, leading to poor prognosis. In recent years, many efforts have been devoted to investigating potential new and more effective drugs and, indeed, the treatment armamentarium for advanced HCC has broadened tremendously, with targeted- and immune-therapies, and probably the combination of both, playing pivotal roles. Together with new established knowledge, many issues are emerging, with the role of neoadjuvant/adjuvant settings, the definition of the best transitioning time from loco-regional treatments to systemic therapy, the identification of potential predictive biomarkers, and radiomics being just some of the topics that will have to be further explored in the next future. Clearly, the current COVID-19 pandemic has influenced the management of HCC patients and some considerations about this topic will be elucidated.

## 1. Introduction

Hepatocellular carcinoma (HCC) accounts for more than 90% of cases of non-metastatic tumors of the liver [1]. Risk factors include hepatitis B and C, fatty liver disease, alcohol-related cirrhosis, and different dietary exposures [2].

Chronic liver disease causes inflammation, fibrosis, and aberrant cellular regeneration that may favor genetic and epigenetic modification as the basis of dysplasia. Mutations in the TERT promoter and in the TP53 gene and the WNT signaling pathway are very common in HCC [1,3]. Angiogenesis plays a key role in HCC carcinogenesis [4]. At the molecular level, angiogenesis results from an imbalance between promotors and inhibitors of vessel growth and maturation [5]. Proangiogenic factors activate endothelial cell tyrosine kinases and subsequent downstream intracellular signaling through PI3K/Akt/mTOR pathways leading to angiogenesis [6]. In fact, HCC is characterized by an excess of angiogenic factors produced by tumor cells, vascular endothelial cells, immune cells, and the surrounding tumor microenvironment. One of the most important mediators in HCC pathogenesis is vascular endothelial growth factor (VEGF), responsible for abnormal vascular structure and function resulting in hypoxic environment. The hypoxic conditions further stimulate other angiogenic factors that promote cell proliferation and autophagy. Autophagy generates energy for cancer cells and therefore promotes cancer progression [7,8]. In fact, hypoxia is presumed to robustly stimulate tumor angiogenesis [9], and, therefore, currently approved treatments for advanced HCC in first- and second-line settings target angiogenic pathways. Liver resection, ablation, and liver transplantation are potentially curative but require diagnosis at a sufficiently early stage. However, up to 80% of HCC are diagnosed in an advanced stage when systemic therapies remain the main option [10]. The treatment efficacy and the prognosis of advanced HCC patients remain poor, the median survival following diagnosis ranges approximately between 6 and 20 months [11].

It is important to consider that most of HCC patients present at the diagnosis with underlying liver cirrhosis and compromised liver function that necessarily have consequences on feasibility and tolerance of cancer treatments [12]. In fact, survival in HCC patients depends not only on tumor stage, but also on underlying liver function, graded according to the widely used Child–Pugh system [13]. Evidence shows that sorafenib efficacy and clinical outcomes are correlated with Child–Pugh stage (CPs)—patients with CPs B have a worse outcome than those with CPs A [14,15,16].

Liver function and cancer-related symptoms must be considered to predict survival and impact of treatment.

In this review, we describe the current treatment landscape of advanced HCC, covering the spectrum from the most-known sorafenib to the newest target- and immune-therapies. We also explore concepts that are progressively gaining more attention, such as the neoadjuvant and adjuvant setting, the need to define the transitioning time from loco-regional treatments to systemic therapy, and potential predictive biomarkers of available drugs and radiomics. Lastly, considering the current COVID-19 pandemic, we include some considerations about its impact on the treatment of HCC.

## 2. Target Therapies

**Figure 1:**
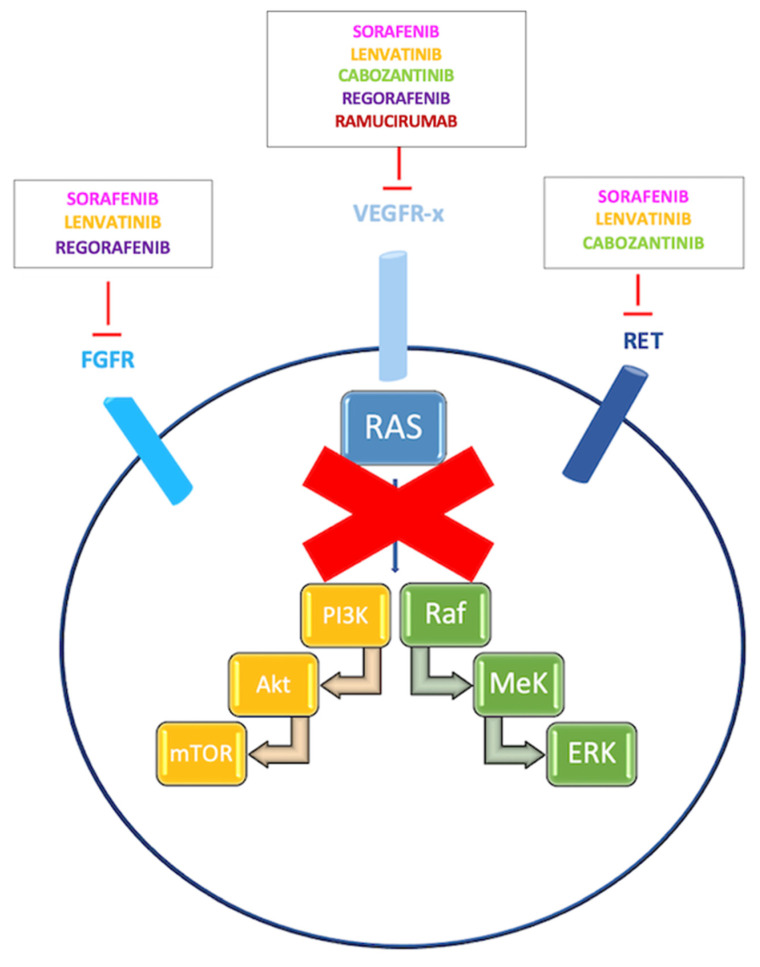
Inhibition of targets VEGFR, fibroblast growth factor receptor (FGFR) and RET by sorafenib, lenvantinib, regorafenib, cabozantinib, and ramucirumab.

HCC develops on the basis of multiple biological processes, including genetic and epigenetic alterations [17,18]. For more than ten years, sorafenib has represented the only systemic treatment with clinical efficacy for patients diagnosed with unresectable HCC. Recently, scenario has dramatically changed and several other target therapies such as lenvatinib, regorafenib, cabozantinib, and ramucirumab have been shown to be superior to placebo after sorafenib failure (see also Figure 1) [18,19].

### 2.1. First-Line of Treatment

#### 2.1.1. Sorafenib

Sorafenib is a tyrosine-kinase inhibitor (TKI) that blocks tyrosine kinase receptors, VEGFR-2 and 3, PDGFR-β, c-Kit, FLT-3, fibroblast growth factor receptor (FGFR), and RET), downstream pathway kinases (Ras/Raf/MEK/ERK, JAK/STAT), and other targets (c-Raf, B-Raf) [20,21,22]. From 2007, oral administration of sorafenib has been recommended worldwide as the first-line therapy for advanced stages of HCC [23]. According to the SHARP trial (sorafenib therapy in advanced hepatocellular carcinoma), which enrolled 602 advanced HCC patients, sorafenib at the dosage of 400 mg twice-daily showed a 2.8 month prolonged survival for HCC patients treated with sorafenib when compared to the placebo group. Median overall survival (mOS) was 10.7 months in the sorafenib group versus 7.9 months in the placebo group [23].

After approval of sorafenib in this disease setting, many other TKIs were studied in different trials (sunitinib [24], brivanib [25], sorafenib plus erlotinib [26], linifanib [27], cediranib [28], and dovitinib [29]) though only lenvatinib showed non-inferiority compared to sorafenib in first-line setting [30].

#### 2.1.2. Lenvantinib

Lenvatinib is an oral TKI that blocks activities of VEGFR1–3, fibroblast growth factor receptors (FGFRs) 1–4, PEGFR, RET, and KIT [31,32]. Lenvatinib was approved for the first-line therapy of advanced HCC following the results of the REFLECT trial, a randomized phase III non-inferiority trial [30]. The REFLECT trial enrolled 954 patients and compared the efficacy of lenvatinib and sorafenib for first-line treatment of patients with unresectable HCC. The median OS was 13.6 months in the lenvatinib group compared to 12.3 months in the sorafenib group. The dosage of lenvatinib was 12 mg/day for bodyweight ≥ 60 kg or 8 mg/day for bodyweight <60 kg. Treatment efficacy and serum levels of AFP were explored, showing that patients with AFP (alpha feto protein) serum level >200 ng/mL and treated with lenvatinib had a significantly longer OS (10.4 vs. 8.2 months) [30].

### 2.2. Subsequent Lines of Treatment

From second-line (included) to further lines of treatment.

#### 2.2.1. Regorafenib

Regorafenib is a small multi-target inhibitor of VEGFR1, TIE-2, RETRAF-1, BRAF, PDGFR, and FGFR [32]. The RESORCE trial, a randomized phase III trial, demonstrated the effectiveness of regorafenib in HCC patients who experienced disease progression on sorafenib treatment [33]. Interestingly, the efficacy of regorafenib was independent from the rate of disease progression during prior sorafenib treatment or the last sorafenib dose [34,35]. The RESORCE trial demonstrated a statistically significant improvement in mOS in patients who received regorafenib after sorafenib treatment (10.6 in the experimental arm versus 7.8 months in the placebo group) [33].

#### 2.2.2. Cabozantinib 

Cabozantinib is an oral TKI that blocks the activities of VEGFR 1-3, c-MET, AXL, and the angiopoietin receptors TIE-2, RET, c-Kit, and FLT-3 [36]. Its approval is based on the CELESTIAL trial (cabozantinib vs placebo in subjects with HCC who have received prior sorafenib) [37], a randomized phase III clinical study where cabozantinib, at a dosage of 60 mg once daily, was challenged as a second-line therapy for advanced HCC [38]. Cabozantinib achieved a mOS of 10.2 months versus 8.0 months for the placebo group [37].

#### 2.2.3. Ramucirumab

Ramucirumab is a fully-human monoclonal antibody (IgG1) that suppresses angiogenesis by binding to VEGFR2. It was studied in the REACH trial, a randomized, phase III trial [39], investigating the efficacy of ramucirumab versus placebo as a second-line treatment in 565 patients with advanced HCC after first-line therapy with sorafenib. The dosage of ramucirumab was 8 mg/kg every two weeks. In the REACH trial, ramucirumab failed to significantly increase OS versus placebo in unselected patients [39]. However, the subgroup analysis demonstrated a survival benefit in HCC patients with high serum levels of AFP (400 ng/mL or higher) [36,38,40]. The subsequent phase III REACH-2 trial, confirmed this result, leading to the approval of ramucirumab as second-line treatment for advanced HCC patients with high AFP. In particular, mOS on the ramucirumab arm was 8.5 months versus 7.3 months in the placebo group [41].

## 3. Immune Checkpoint Inhibitor (ICI) Monotherapy

In the last 10 years, immune-oncology (IO) has represented a major breakthrough in anticancer therapy in some malignancies (especially non small-cell lung cancer and melanoma) [42]. It has been demonstrated that HCC develops in an inflammatory microenvironment and that immune tolerance plays a central role in hepatocarcinogenesis [43,44]. Immune check-point inhibitors (ICI) of the programmed cell death protein 1 or ligand 1 (PD-1/PD-L1) or of the cytotoxic T-lymphocyte-associated antigen 4 (CTLA-4) are the most-studied immunotherapeutic agents. The two pathways play their role at different stages of the immune response. The PD-1/PD-L1 pathway suppresses T-cell activity and mediates the differentiation of regulatory T cells, thus promoting immune tolerance, while the CTLA-4 pathway inhibits the potential proliferation of autoreactive T cells, thus preventing autoimmunity [45,46]. ICIs can therefore revert immune tolerance to neoplastic cells, thus enhancing their immune-mediated elimination [47].

Clinical trials with the most mature data have been conducted with the anti-programmed cell death protein-1 (PD-1) immune checkpoint inhibitors nivolumab, a fully human immunoglobulin (Ig) G4 (IgG4) mAb, and pembrolizumab, a humanized anti-PD-1 IgG4 mAb.

### 3.1. Anti-PD-1

#### 3.1.1. First-Line of Treatment

Checkmate 040 was the pivotal, phase I/II, non-comparative, dose escalation, and expansion trial study that tested the safety and efficacy of nivolumab in advanced sorafenib-naïve or sorafenib-experienced hepatitis B virus (HBV), hepatitis C virus (HCV), or uninfected HCC. It demonstrated a favorable response rate of 20% in the dose-expansion phase and of 15% in the dose-escalation phase, with a manageable safety profile [48].

Based on Checkmate 040, the Food and Drug Administration (FDA) granted an accelerated approval to nivolumab for the second-line line treatment of HCC (September 2017) [49].

The subsequent phase III Checkmate 459 compared nivolumab with sorafenib as first-line treatments for unresectable HCC. Median OS was 16.4 months in the nivolumab arm and 14.7 months in the sorafenib arm of the trial, but the protocol-defined statistical significance threshold for OS was not met (HR = 0.85, 95% CI: 0.72–1.02, *p* = 0.0752) [50]. Safety profile at the long-term follow up was consistent with that of the primary analysis and nivolumab continued to demonstrate a more favorable safety profile than sorafenib, with fewer grade 3-4 treatment-related adverse events (TRAEs) (22% vs. 50%) and fewer TRAEs leading to discontinuation [51].

The novel IgG4 anti-PD-1 mAb tislelizumab, able to potentially overcome one of the anti-PD-1 therapy resistance mechanisms, the so-called antibody-dependent cellular phagocytosis (ADCP) [52], was investigated in a phase I trial showing an objective response rate (ORR) of 12.2% and a disease control rate (DCR) of 51.0% with a favorable safety profile [53]. Prompted by these encouraging results, tislelizumab is currently being further evaluated in the phase III trial RATIONALE 301, comparing tislelizumab to sorafenib for the first-line treatment of advanced HCC patients [54].

#### 3.1.2. Second-Line of treatment

Keynote-224 is a non-randomized phase II study that evaluated the safety and efficacy of pembrolizumab for the second-line treatment of 104 sorafenib-experienced advanced HCC patients. An objective response (OR) was observed in 17% of patients, including one complete response (CR) and 17 partial responses (PR). Median progression-free survival (mPFS) and mOS were, respectively, 4.9 months (95% CI 3.4–7.2) and 12.9 months (95% CI 9.7–15.5) and 1-year OS rate was 54% (95% CI 44–63) [55]. Based on the Keynote-224 study, the FDA granted an accelerated approval to pembrolizumab for the second-line treatment of HCC (November 2018) [56].

Pembrolizumab was then tested in sorafenib-progressors or intolerant HCC patients in the phase III, randomized, placebo-controlled Keynote-240. The study showed an improvement in the two co-primary endpoints of OS (HR = 0.78, 95%CI: 0.61–0.99, *p* = 0.0238) and PFS (HR = 0.78, 95%CI: 0.61–0.99, *p* = 0.0209); however, it did not meet its pre-specified threshold for statistical significance [57].

### 3.2. Anti-PD-L1

Regarding anti-PD-L1 ICIs, durvalumab has been tested in a phase I/II trial in HCC patients pre-treated with sorafenib, showing an ORR of 10.3% [58]. In the same setting of patients, avelumab is being evaluated in an ongoing phase II study [59].

Despite being not statistically significant in terms of OS, data regarding the improvement of OS suggest that ICIs are active in HCC and support their role for the treatment of advanced HCC. Clearly, it is crucial to identify the group of patients with the highest chance of reaching clinical benefit from IO. Strategies have been proposed, with PD-L1 expression, tumor mutational burden (TMB), and gut microbiota above all, but their clinical validation is warranted [60].

Another strategy that is being explored to further enhance ICI’s potentiality is the dual immune checkpoint blockade or the combination of ICIs with other classes of therapeutic agents.

## 4. Chemotherapy

Preliminary studies suggest that HCC patients may benefit from metronomic capecitabine as a second-line treatment after sorafenib progression.

Capecitabine is an oral precursor of 5-fluorouracil (5-FU) and its metronomic use has been introduced in the recent years. Safety and efficacy of metronomic capecitabine in HCC patients were evaluated by Casadei Gardini et al. [61], suggesting a possible activity of capecitabine (versus best supportive care, BSC) and a tolerable toxicity profile.

Similar results were obtained by Trevisani et al. [62] in 2018, showing the efficacy and safety of capecitabine for the second-line systemic therapy of HCC patients.

## 5. Combination Therapies

In the last ten years, the good/promising results obtained by employing target- and immune-therapies for advanced HCC have definitely changed research priorities.

Currently, research is mostly focusing on ICIs, anti-VEGF, and TKI combination therapies, by means of their potential synergism and interplay to improve outcomes and benefit of HCC patients. Clinical data are definitely encouraging this approach.

### 5.1. ICIs + ICIs

The use of combinations of ICIs is expected to be a promising strategy for the treatment of HCC patients; in particular, most of the studies are focusing on the use of anti-PDL-1/PD-1 combined with anti-CTLA-4 antibodies. The biological basis for this combination relies on the fact that the inhibition of the CTLA-4 pathway produces a proliferation of CD8+ cells in the lymph nodes and in the tumor tissue that is critical to stimulate the immune response mediated by anti-PDL-1/PD-1 antibodies [63,64].

The Checkmate 067 phase III clinical trial showed the superiority of the combination of the anti-PD-1 nivolumab and the anti-CTLA-4 ipilimumab over nivolumab in monotherapy, prolonging OS in advanced melanoma patients [65].

One of the Checkmate 040 cohort is currently evaluating safety and tolerance of the same combination therapy (nivolumab and ipilimumab) in sorafenib pre-treated advanced HCC patients; results of this trial are awaited, but preliminary data showed good responses and a favorable safety profile for the combination [48,66].

The combination of durvalumab (anti-PDL-1) and tremelimumab (anti-CTLA-4) for HCC patients was tested in a phase I/II clinical trial and the results, published in 2017, indicated promising benefits from the use of this combination strategy [67]. On the basis of the encouraging results, the HIMALAYA study (study of Durvalumab and Tremelimumab as first-line treatment in patients with advanced Hepatocellular Carcinoma), a randomized, multicenter, phase III study was designed and is currently ongoing to assess the efficacy of durvalumab plus tremelimumab as first-line treatment for patients with unresectable HCC [68].

### 5.2. ICIs + Target Therapies

Combination regimes of ICIs and agents that target specific pathways involved in tumor growth have been developed to improve ICI efficacy.

### 5.3. ICIs + anti-VEGF

The use of antiangiogenic therapies combined with ICIs is a therapeutic strategy with a strong biological rationale, supported by preclinical evidence. The key to this approach is the confirmed role of hypoxia in the regulation of immune response.

Hypoxia of solid tumors and the resulting production/secretion of hypoxia-inducible factor-1alpha (HIF-1a) cause the up-regulation of PDL-1 expression in tumor-infiltrating myeloid cells (in particular in myeloid-derived suppressor cells, MDSCs). In this context, VEGF, a signal protein stimulating the formation of blood vessels whose release is stimulated by HIF in a condition of hypoxia, acts as an immunosuppressive molecule PDL-1 blockade in hypoxic microenvironment enhances MDSC-mediated T-cell activation and reduces the production of cytokines (IL-6 and IL-10). Hypoxia can be induced by antiangiogenetic therapies; simultaneous blockade of PDL-1 and HIF-1alfa (that generate hypoxia) may have synergistic effects [69,70].

Several trials exploring combinations of antiangiogenics and ICIs in HCC are ongoing.

Pishvaian et al. showed the results of the phase Ib study of atezolizumab and bevacizumab in HCC; ORR was 32% in the 73 evaluable patients, the combination therapy was generally well tolerated, the safety profile was manageable, and responses were durable [71].

The promising efficacy and safety of the phase Ib trial led to the design of IMbrave150, a multicenter phase III ongoing trial [72]. Preliminary data showed an OS at 12 months of 67.2% and median PFS of 6.8 months for patients treated with combination therapy versus 54.6% and 4.3 months, respectively, for patients treated with sorafenib. These data confirmed a statistically significant and clinically meaningful improvement in OS and PFS for atezolizumab and bevacizumab (versus sorafenib), making this combination therapy a promising and likely practice-changing first-line therapy for HCC patients in the future.

### 5.4. ICIs + TKI

Many clinical trials have tested the immunomodulatory profile of TKI associated with ICIs in different subset of oncological patients and preliminary data have shown promising efficacy and safety [73]. On the basis of the good results of the phase III CELESTIAL trial, showing an OS improvement for patients treated with cabozantinib versus placebo [37], the COSMIC-312 study, evaluating the combination of cabozantinib and atezolizumab, was designed [74]. Preclinical and clinical data showed promising results for the combination of cabozantinib plus atezolizumab, which may have a synergistic effect and a manageable safety profile [75,76]. The ongoing COSMIC-312 study will evaluate PFS, OS and safety for the combination therapy and may define the role of this association as a first-line treatment option for HCC.

Furthermore, the combination of lenvatinib and pembrolizumab has been studied in recent years. Preclinical studies revealed that lenvatinib has an increased anti-tumor activity when combined with PD-1 inhibitors rather than when it is used in monotherapy [77].

Press releases of the phase Ib trial of lenvatinib and pembrolizumab for the first-line treatment of HCC showed a radiological response rate of 46% in the 13 evaluable patients, with a manageable toxicity profile [78]. On the basis of these encouraging results, a phase III multicenter clinical trial (LEAP-002 TRIAL) is ongoing to assess efficacy and safety of lenvatinib plus pembrolizumab compared to lenvatinib plus placebo in advanced HCC [79].

## 6. Defining the Best Sequencing Strategy

**Figure 2:**
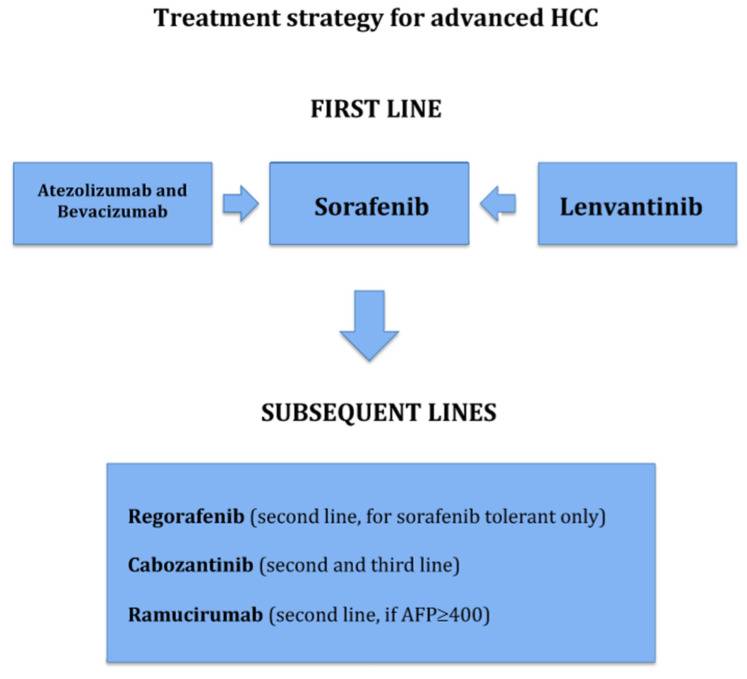
Treatment strategy for advanced HCC: possible therapeutic sequencing. First-line: sorafenib, lenvantinib, or the combination atezolizumab/bevacizumab. Subsequent lines: regorafenib, cabozantinib, or ramucirumab.

To summarize what has been elucidated in the previous paragraphs, Figure 2 defines a possible sequencing strategy for HCC treatment for first and subsequent lines of treatment. Ongoing phase III trials are summarized in Table 1.

## 7. Unmet Needs and Future Perspectives

### 7.1. Neoadjuvant/Adjuvant Setting

Liver transplantation, surgical resection, and ablative therapies are the only curative-intent opportunities for HCC patients. However, these options are in most cases unfeasible because of the frequent advanced chronic liver disease and compromised liver function. Therefore, recent studies focused on the possible role of neoadjuvant and adjuvant therapies for HCC patients, in order to make a larger number of patients eligible for to surgery and to decrease the risk of recurrent disease [80].

The use of sorafenib in the neoadjuvant management of advanced HCC has been reported in few isolated cases; larger clinical experience is still lacking.

Barbier et al. described the cases of two advanced HCC patients treated with sorafenib for nine months who gained a good clinical and radiological response and were subsequently treated with surgery with curative results [81]. Irtan et al. reported two similar cases of locally advanced HCC that achieved complete regression after neoadjuvant therapy with sorafenib [82]. The BIOSHARE study (results of pre-operative sorafenib in patients with resectable HCC), an open-label multicenter phase II study designed to evaluate the activity of neoadjuvant sorafenib in patients with resectable HCC, is obtaining encouraging results; all patients underwent tumor resection, R0 was achieved in 88% of cases and sorafenib toxicity profile was favorable [83].

The other TKI, ICI, and VEGF inhibitors have not been investigated in the neoadjuvant setting.

TACE (transarterial chemoembolization) is a widely used first line treatment for unresectable HCC treatment; it is based on the embolization of the arterial blood that supplies the tumor and the injection of chemotherapy. Notably, the efficacy of TACE in the neoadjuvant setting has been explored; a retrospective review of 1457 patients who underwent hepatectomy showed an advantage in terms of disease-free survival (DFS), in particular in patients treated with two TACE sessions [84]. Favorable results in median OS and relapse-free survival (RFS) were found in research by Li et al. [85]. Nevertheless, no differences in DFS were seen in the comparative analysis made by Sasaky et al. [86] of patients who underwent TACE and those who did not. More trials are definitely required to better evaluate the efficacy of preoperative TACE.

On the basis of the promising data regarding the use of neoadjuvant therapy, it may be interesting to select patients in good general condition for systemic treatment as a bridge to surgery, in order to facilitate downstaging of unresectable patients and to increase the number of surgical candidates.

The incidence of recurrent disease following surgical resection is around 50% at three years and 70% at five years in HCC patients [1]. Many studies have evaluated the possibility of introducing adjuvant systemic therapy to reduce the risk of relapse and prolong survival after surgery, but effective use of these therapies is controversial in clinical practice and there is not a standard-of-care [87]. Adjuvant interferon (INF) therapy resulted in improved RFS and OS, but its toxicity profile was not favorable [88].

The STORM (adjuvant sorafenib for HCC after resection or ablation) phase III randomized study of sorafenib versus placebo as adjuvant treatment did not show significant differences between the two groups in terms of RFS, time to recurrence, or OS with increased adverse effects in the treatment arm [89]. Promising data are emerging from studies with immunotherapy; postoperative immunotherapy seemed to prevent recurrence after radical resection, without OS improvement [90,91,92].

Currently, some trials are ongoing to assess the role of immunotherapy after liver surgery. In particular, KEYNOTE-937 is evaluating the safety and efficacy of pembrolizumab versus placebo in adjuvant setting [93] and CheckMate 9DX is testing adjuvant nivolumab versus placebo in a phase III, randomized, double-blind study [94].

It will be interesting to evaluate if the results obtained in the advanced setting can be translated in the adjuvant context.

### 7.2. Timely Transitioning from Loco-Regional to Systemic Treatments

TACE is the first-line treatment for HCC at intermediate stage; it confers survival benefits with few adverse effects. However, it may happen that patients with multiple nodules and large-sized HCC undergo repeated TACE without therapeutic advantages and with many adverse effects that increase liver damage and impair the hepatic functional reserve. The most-employed definition of TACE refractoriness/failure, even though not universally employed, has been given by the Japan Society of Hepatology: TACE failure is defined as an insufficient response after two or more consecutive TACE procedures [95,96].

Two different studies evaluated the possibility of switching to sorafenib after TACE failure; both the research groups showed that the early employment of systemic therapy give patients improved survival benefits compared to loco-regional treatments, strongly supporting the conversion to sorafenib despite the intermediate stage of disease [97,98]. Kudo M. confirmed those results [99].

It is important to define the best moment for the transition from loco-regional to systemic treatment when HCC patients are unresponsive to TACE in order to initiate systemic therapy as early as possible and maximize therapeutic effects.

### 7.3. Predictive Biomarkers

Predictive biomarkers are widely adopted to predict response to cancer treatment and they are considered the key for the successful development of new drugs. Currently, however, there are no validated predictive biomarkers for HCC and their identification is compelling.

As already discussed, sorafenib is the first line of treatment for patients with advanced HCC; notwithstanding its large use, there is still a lack of validated predictive biomarkers that could identify who would benefit from sorafenib treatment and rule out resistant patients. Feng et al. [100], on the basis of previous studies [101,102], investigated the possible role of ACSL4, an activating enzyme of ferroptosis, the iron-dependent type of programmed cell death essential for sorafenib-induced cytotoxicity on HCC cells The study suggested the possible role of this protein in predicting sorafenib sensitivity of HCC cells both in vitro and in vivo; 66.7% of responders had higher ACSL4 expression.

The predictive value of pERK is uncertain; in fact, even if in a phase II study its levels were correlated with longer time to progression (TTP), these results were not confirmed by subsequent trials [103,104].

Wang et al. recently published their data about the role of PI16, a gene with differential expression in many tumor cells; low levels of PI16 were significantly associated with longer PFS and OS in patients treated with sorafenib, suggesting its possible value as a predictive biomarker [105].

The role of microRNA as a prognostic factor was previously described; there are a few studies that have evaluated the possible association between miRNA and disease control. In particular, Nishida et al. [106] evaluated the predictive role of miR-181a-5p level before treatment and Kohno et al. [107] evaluated the role of has-miR-30. Both the studies assumed, on the basis of their results, that miRNA might serve as predictive factor for sorafenib therapy.

Recently, the value of single-nucleotide polymorphisms on angiopoietin-2 (ANGPT1) and endothelial-derived nitric oxide synthase (NOS) genes was evaluated in patients with advanced HCC treated with sorafenib. Both univariate and multivariate analyses identified polymorphisms of these genes correlated to resistance to therapy [108].

In conclusion, after more than 10 years of research into sorafenib, the finding of validated predictive biomarkers of response in HCC still represents an unmet need; in the future, an alternative approach may be the use of metabolomic profiling and genomic analysis in order to identify those patients who are more likely to benefit from sorafenib therapy [109].

Predictive biomarkers of response to lenvatinib therapy are an object of study. Considering the mechanism of action of lenvatinib, a recent study tested the role of circulating angiogenic factors (CAFs). Serum levels of VEGF, FGF19, FGF23, and Ang-2 were measured and, in lenvatinib responders, a significant correlation between increased level of FGF19 and decreased levels of Ang-2 was found [110].

### 7.4. Radiomics

Radiomics is an emerging field in medical imaging, potentially leading to a change in routine clinical practice in the near future. Through the extraction of quantitative features from conventional imaging exams (computed tomography and magnetic resonance imaging), by mean of computer-based algorithms radiomics correlates these features with meaningful clinical endpoints, eventually leading to a personalized clinical approach [111]. The spectrum of radiomics’ applications is broad, with diagnosis and prognosis estimation above the primarily investigated. [112]. Radiomics, whether it complements or replaces tumor biopsy, can identify HCC with high-risk features and this might be extremely important in cases where no tissue sampling is available. Moreover, being correlated with HCC biological characteristics, radiomics features might represent imaging biomarkers with predictive and prognostic significance, paving the way for personalized treatment [113,114].

## 8. Impact of COVID-19 Pandemic at a Glance

As no one would ever have imagined, in 2020, a new pandemic undoubtedly put societies all over the world to the test. The 2020 SARS-CoV-2 outbreak and the related severe pneumonia has had a significant impact on healthcare organizations and on the general management of oncological patients that have a higher risk of infections [115]. In most countries, oncological centers remodulated access to the hospital and cancer management in order to minimize the exposure of cancer patients to the infection. COVID-19 emergency caused delays in cancer diagnosis, deferment in of scheduled surgery, and modifications of follow-up programs [116,117]. HCC patients are a very frail category among cancer patients. SARS-CoV-2 infection may cause hepatic injury that exacerbates chronic liver disease, thus hampering the delicate HCC patients’ balance and generating problems in cancer treatments [106]. For early stage HCC, radiofrequency and microwave ablation were preferred (when possible) to surgery because they were more accessible and had a shorter waiting time. In the context of advanced stage of disease treated with oral TKI, in order to reduce the number of cancer patients accessing the hospital, visits were scheduled with longer intervals and management of toxicities was undertaken by the family doctor or by telephone consultations [118]. Some questions about ICI therapies and susceptibility to COVID-19 infection are still open. It is certainly important to provide a continuum of care for oncological patients even during the COVID-19 pandemic and to re-organize hospital activity in order to guarantee the best treatment options and, at the same time, to protect patients and minimize their risk of infection.

## 9. Conclusions

In summary, advanced HCC treatment has considerably improved in recent years; novel drugs have proved to be effective, thus underscoring the importance of defining the best sequencing strategy, and more are currently being studied. Together with the progressively growing advances, researchers are also facing unmet needs regarding diagnosis, treatment, and prognosis estimation, and these will have to be addressed in the near future. Importantly, HCC patients will always have to be placed at the center of the continuum of care, even in the current COVID-19 pandemic era.

## Figures and Tables

**Table 1:** Ongoing phase III clinical trials in different treatment setting (www.clinicaltrial.gov).

	Status	Clinical Trial	Phase	Treatment Setting
NCT04102098	Recruiting	A study of atezolizumab plus bevacizumab versus active surveillance as adjuvant therapy in patients with hepatocellular carcinoma at high risk of recurrence after surgical resection or ablation	Phase III	Adjuvant
NCT03867084	Recruiting	Safety and efficacy of pembrolizumab (MK-3475) versus placebo as adjuvant therapy in participants with hepatocellular carcinoma (HCC) and complete radiological response after surgical resection or local ablation (MK-3475-937/KEYNOTE-937)	Phase III	Adjuvant
NCT03859128	Recruiting	Toripalimab or placebo as adjuvant therapy in hepatocellular carcinoma after curative hepatic resection	Phase II/ III	Adjuvant
NCT03847428	Recruiting	Assessment of efficacy and safety of durvalumab alone or combined with bevacizumab in high-risk-of-recurrence HCC patients after curative treatment	Phase III	Adjuvant
NCT03383458	Recruiting	A study of nivolumab in participants with hepatocellular carcinoma who are at high risk of recurrence after curative hepatic resection or ablation	Phase III	Adjuvant
NCT02738697	Recruiting	Adjuvant chemotherapy with FOLFOX in HCC patients after resection	Phase III	Adjuvant
NCT03778957	Recruiting	A global study to evaluate transarterial chemoembolization (TACE) in combination with durvalumab and bevacizumab therapy in patients with locoregional hepatocellular carcinoma	Phase III	Locoregional disease
NCT04523493	Recruiting	Phase III study of toripalimab (JS001) combined with lenvatinib for advanced HCC	Phase III	Palliative
NCT04246177	Recruiting	Evaluation of safety and efficacy of lenvatinib (E7080/MK-7902) with pembrolizumab (MK-3475) in combination with transarterial chemoembolization (TACE) in participants with incurable/non-metastatic hepatocellular carcinoma (MK-7902-012/ E7080-G000-318/LEAP-012)	Phase III	Palliative
NCT04039607	Recruiting	A study of nivolumab in combination with ipilimumab in participants with advanced hepatocellular carcinoma	Phase III	Palliative
NCT03794440	Recruiting	A study to evaluate the efficacy and safety of sintilimab in combination with IBI305 (anti-VEGF monoclonal antibody) compared to sorafenib as the first-line treatment for advanced hepatocellular carcinoma.	Phase II/III	Palliative
NCT03764293	Recruiting	A study to evaluate SHR-1210 in combination with apatinib as first-line therapy in patients with advanced HCC	Phase III	Palliative
NCT03755791	Recruiting	A study of cabozantinib in combination with atezolizumab versus sorafenib in subjects with advanced HCC who have not received previous systemic anticancer therapy	Phase III	Palliative

## Data Availability

No available date.
